# The multimodal antidepressant vortioxetine exerts therapeutic effects in male mice which is associated with normalization of hippocampal SIK2/CRTC1-related signaling and require hippocampal CRTC1

**DOI:** 10.3389/fphar.2026.1837566

**Published:** 2026-08-20

**Authors:** Hui Xu, Hua Fan, E-hui Ding, Peng Hou, Bo Jiang, Wei-bing Zhu

**Affiliations:** 1 Department of Neurosurgery, Nantong Hospital to Nanjing University of Chinese Medicine, Nantong, Jiangsu, China; 2 The First Affiliated Hospital, College of Clinical Medicine of Henan University of Science and Technology, Luoyang, Henan, China; 3 Department of Pharmacology, School of Pharmacy, Nantong University, Nantong, Jiangsu, China

**Keywords:** cAMP response element binding protein (CREB), CREB-regulated transcription co-activator 1, depression, hippocampus, salt-inducible kinase 2, vortioxetine

## Abstract

**Introduction:**

The multimodal antidepressant vortioxetine combines serotonin reuptake inhibition with activity at several serotonin receptor subtypes, yet its downstream mechanisms related to neuroplasticity remain incompletely understood. This study investigated whether vortioxetine’s antidepressant efficacy involves hippocampal salt-inducible kinase 2 (SIK2) and CREB-regulated transcription coactivator 1 (CRTC1).

**Methods:**

By using chronic social defeat stress (CSDS), chronic unpredictable mild stress (CUMS), and chronic restraint stress (CRS) models in male C57BL/6J mice, we assessed the effects of vortioxetine (10 and 20 mg/kg) on depressive-like behaviors, hippocampal SIK2 and CRTC1 expression, CRTC1 subcellular localization, and CRTC1-CREB interaction in mice. Viral-mediated hippocampal CRTC1 knockdown was employed to establish causality.

**Results:**

Vortioxetine dose-dependently reversed chronic stress-induced behavioral deficits and concurrently normalized hippocampal SIK2 upregulation and CRTC1 downregulation at both transcriptional and translational levels. Furthermore, vortioxetine restored nuclear CRTC1 translocation and enhanced CRTC1-CREB binding. Critically, hippocampal CRTC1 knockdown completely abolished the behavioral benefits of vortioxetine.

**Discussion:**

These findings demonstrate that vortioxetine’s antidepressant actions in male mice are associated with normalization of hippocampal SIK2/CRTC1-related signaling and require hippocampal CRTC1, providing a novel molecular mechanism underlying its multimodal efficacy.

## 1 Introduction

Major depressive disorder (MDD) represents a leading cause of global disability, imposing substantial personal suffering and socioeconomic burden (Thom et al., 2019). Despite decades of research and therapeutic advances, the pathophysiological underpinnings of MDD remain incompletely understood (Dean and Keshavan, 2017; Ménard et al., 2016). The prevailing monoaminergic hypothesis, which posits a central deficiency in serotonin (5-HT) and norepinephrine neurotransmission, has guided the development of most first-line antidepressants, including selective serotonin reuptake inhibitors (SSRIs) and serotonin-norepinephrine reuptake inhibitors (SNRIs) (Perez-Caballero et al., 2019). However, the significant limitations of these conventional agents—notably, delayed onset of action (weeks to months), low remission rates, and residual cognitive and emotional symptoms in many patients—underscore the complex, multi-faceted nature of depression and highlight an urgent need for treatments with novel mechanisms of action (Boku et al., 2018; Gonda et al., 2023).

Contemporary models of depression increasingly emphasize disruptions in neuroplasticity and cellular resilience beyond mere neurotransmitter deficits (Mudgal et al., 2022; Price and Duman, 2020). Convergent evidence implicates dysfunction within the hippocampus, a brain region critical for mood regulation and cognitive function, as a central node in MDD pathology (Tartt et al., 2022). Chronic stress, a major precipitating factor for depression, reliably reduces hippocampal volume, suppresses neurogenesis, and downregulates the brain-derived neurotrophic factor (BDNF) signaling cascade (Castrén and Monteggia, 2021; Gao et al., 2025; Parul et al., 2021; Sheline et al., 2019). BDNF, in turn, is a key effector of synaptic plasticity and neuronal survival, and its expression is transcriptionally regulated by the cAMP response element-binding protein (CREB) (Amidfar et al., 2020; Lu et al., 2014). The activity of CREB is modulated by CREB-regulated transcription coactivators (CRTCs), whose nuclear translocation and subsequent CREB binding are inhibited through phosphorylation by salt-inducible kinases (SIKs) (Darling and Cohen, 2021; Yin et al., 2022; Yoon et al., 2021). Compelling preclinical work has demonstrated that chronic stress upregulates hippocampal SIK2 expression, leading to CRTC1 phosphorylation, cytoplasmic sequestration, and a consequent suppression of CREB-mediated BDNF transcription (Cai et al., 2024; Jiang et al., 2019). Conversely, genetic or pharmacological inhibition of hippocampal SIK2 promotes CRTC1 nuclear translocation, restores BDNF signaling, and exerts robust antidepressant-like effects (Huang et al., 2023; Jiang et al., 2019; Liu et al., 2020), positioning hippocampal SIK2 and CRTC1 as feasible therapeutic targets.

Vortioxetine, a multimodal antidepressant approved in 2013, represents a distinct pharmacological approach that extends beyond simple serotonin transporter (SERT) inhibition (Sanchez et al., 2015). Its unique receptor activity profile—comprising 5-HT_1A_ agonism, 5-HT_1B_ partial agonism, and 5-HT_1D_, 5-HT_3_, and 5-HT_7_ antagonism—confers dose-dependent clinical effects by engaging successive receptor targets with increasing doses (Krupa et al., 2023; Sowa-Kućma et al., 2017). Notably, 5-HT_3_ antagonism disinhibits noradrenergic and dopaminergic transmission in the prefrontal cortex (PFC) and reward system, which may reduce emotional blunting and anhedonia. Concurrently, 5-HT_1A_ agonism and 5-HT_7_ antagonism synergistically accelerate 5-HT_1A_ auto-receptor desensitization, enhancing serotonergic transmission and boosting PFC dopaminergic activity, actions that are thought to promote anxiolytic, pro-cognitive, and hedonic effects while mitigating sexual dysfunction. Additionally, 5-HT_1B_ partial agonism reinforces antidepressant and anxiolytic efficacy and may counteract weight gain. For now, the role of 5HT_1D_ antagonism is unclear.

Up to date, emerging preclinical data suggest that vortioxetine’s actions extend to enhancing synaptic plasticity and increasing the expression of plasticity-related genes in the hippocampus (Kugathasan et al., 2017; Li et al., 2023; Waller et al., 2017a; Waller et al., 2017b; Wang et al., 2021c). These findings raise the intriguing possibility that vortioxetine’s clinical advantages may stem, in part, from its engagement with fundamental neuroplasticity pathways. Based on the established role of hippocampal SIK2 and CRTC1 in depression pathophysiology and the demonstrated pro-plasticity effects of vortioxetine, we hypothesized that vortioxetine’s antidepressant mechanism involves modulation of the two molecules. Specifically, we propose that vortioxetine may counteract stress-induced SIK2 hyperactivity, thereby derepressing CRTC1-CREB signaling in the hippocampus. This study aims to systematically test this hypothesis using established mouse models of depression, behavioral pharmacology, and molecular analyses to elucidate whether hippocampal SIK2 and CRTC1 participate in the therapeutic action of vortioxetine.

## 2 Materials and methods

### 2.1 Ethical statement

All experimental procedures were conducted and reported in strict accordance with the ARRIVE 2.0 guidelines for animal research (Percie du Sert et al., 2020). The study protocol was reviewed and approved by the Institutional Animal Care and Use Committee of Nantong Hospital to Nanjing University of Chinese Medicine. Every effort was made to minimize animal numbers and suffering.

### 2.2 Animals

Male C57BL/6J mice (8 weeks old, 22–24 g) and retired male CD1 breeders (more than 9 months old, >40 g) were purchased from SLAC Laboratory Animal Co., Ltd. (Shanghai, China). C57BL/6J mice served as experimental subjects, while CD1 mice were screened for aggression and used as residents in the CSDS paradigm. All the animals were group-housed (except where required by experimental protocols) under the following standard conditions: a 12-h light/dark cycle (lights on at 07:00), controlled temperature (22 °C ± 2 °C) and humidity (55% ± 5%), with *ad libitum* access to food and water. Following 1 week of acclimatization, C57BL/6J mice were weight-stratified and randomly assigned to experimental groups. The sample size for each experiment was determined according to power analysis (G*Power 3.1; α = 0.05; power = 0.80) and many previous studies (Hua et al., 2021; Huang et al., 2020; Jiang et al., 2022; Shi et al., 2022; Su et al., 2019; Wu et al., 2020; Zeng et al., 2020; Zhang et al., 2020; Zhao et al., 2020a; Zhao et al., 2020b). All behavioral tests were performed during the light phase (09:00–17:00) by investigators who were blinded to the treatment conditions. Animals designated for molecular analyses were euthanized via rapid decapitation under brief isoflurane anesthesia (5% induction, 2% maintenance) between 09:00 and 10:00 to minimize circadian variations.

### 2.3 Drug administration

Vortioxetine (TargetMol, Boston, MA, UNITED STATES) solution was prepared fresh daily. Vortioxetine was dissolved in a vehicle consisting of 5% (w/v) dextrose in sterile saline (0.9% NaCl) containing 1% DMSO and 4% Cremophor EL (pH 7.0). Vortioxetine was administered via intraperitoneal (i.p.) injection at a dose of 10 ml/kg body weight. The doses of Vortioxetine (10 and 20 mg/kg) were selected on the basis of previous reports (Li et al., 2023; Wang et al., 2021c). The vehicle control groups received an equivalent volume of the respective vehicle.

### 2.4 Chronic social defeat stress (CSDS)

The CSDS protocol was adapted from established methods (Hernandez Silva et al., 2024; Wang et al., 2021a; Wang et al., 2021b). Briefly, experimental C57BL/6J mice were introduced into the home cage of an unfamiliar and aggressive CD1 resident mouse for 10 min of direct physical interaction. Following the defeat session, the animals were separated by a perforated plexiglass divider, allowing continuous sensory contact for the remainder of the 24-h period. This procedure was repeated for 10 consecutive days, with a different CD1 aggressor each day. To ensure consistency, only CD1 mice that displayed persistent attack latencies of less than 60 s in three consecutive screening sessions were used. Non-stressed control mice were pair-housed with a same-sex conspecifics and handled daily. Drug treatments (vortioxetine or vehicle) were administered once daily for another 2 weeks after CSDS exposure.

### 2.5 Western blotting

According to previous studies (Cai et al., 2024; Huang et al., 2023; Jiang et al., 2019; Liu et al., 2020), hippocampal tissues were rapidly dissected on ice and homogenized in RIPA lysis buffer (Beyotime, Shanghai, China) supplemented with protease and phosphatase inhibitor cocktails (Roche, Basel, Switzerland). Cytoplasmic and nuclear fractions were isolated using a commercial kit (NE-PER, Thermo Fisher, Waltham, UNITED STATES). Protein concentrations were determined by BCA assay. Equal amounts of protein (30 µg) were separated by 10/12% SDS-PAGE and transferred to PVDF membranes (Millipore, Billerica, UNITED STATES). The membranes were blocked with 5% nonfat milk and incubated overnight at 4 °C with the following primary antibodies: anti-SIK2 (1:1,000; Cell Signaling, Danvers, UNITED STATES), anti-CRTC1 (1:1,000; Cell Signaling), anti-phospho-CRTC1 (Ser151) (1:500; Millipore), anti-CREB (1:1,000; Cell Signaling), anti-histone H3 (1:1,000; Cell Signaling), and anti-β-actin (1:5000; Cell Signaling). Following incubation with horseradish peroxidase (HRP)-conjugated secondary antibodies, the bands were visualized using an enhanced chemiluminescence (ECL) substrate (Bio-Rad) and quantified using Image Lab software (Bio-Rad). The data were normalized to those of loading controls (β-actin for total lysates/cytoplasmic fractions and histone H3 for nuclear fractions). Each sample represents an independent biological replicate from a different animal.

### 2.6 Quantitative real-time reverse transcription PCR (qRT-PCR)

According to previous studies (Cai et al., 2024; Huang et al., 2023; Jiang et al., 2019; Liu et al., 2020), after sacrifice, the hippocampal tissues of each C57BL/6J mouse were extracted in cold antifreeze. TRIzol^TM^ reagent (Thermo Fisher) was used to extract RNA, and the RNA concentrations were measured by a Nanodrop 2000 Spectrophotometer (Thermo Fisher). PrimeScript™ RT Master Mix (Takara, Tokyo, Japan) was further used to reverse-transcribe the RNA samples into cDNA which was our PCR template. Afterwards, quantitative PCR was finished by a Step-One-Plus™ Real-Time PCR System (Thermo Fisher) with the SYBR® fast qPCR master mix (Takara). The internal control we adopted was glyceraldehyde-3-phosphate dehydrogenase (GAPDH), and a triplicate assay was performed for each sample. Sangon Biotech (Shanghai, China) provided the PCR primers (listed below). The threshold cycle (CT) was defined as the fractional cycle number at which fluorescence passed the fixed threshold. The relative expression levels of SIK2 mRNA and CRTC1 mRNA were normalized to that of GAPDH and calculated using the △△CT method. Each sample represents an independent biological replicate from a different animal.

**Table:** 

Primers list	Forward	Reverse
SIK2 mRNA	5′-TCAAGGAGCACAAGTGGAT G-3′	5′-TGCATCAGTCGAAGA ACCTG -3′
CRTC1 mRNA	5′-TCTCCGGTCTCCAACCAAG GC-3′	5′-CTGGCTGTCATCTGC TGCTCATAG-3′
GAPDH mRNA	5′-ACATTGTTGCCATCAACGA C-3′	5′-ACGCCAGTAGACTCC ACGAC-3′

### 2.7 Co-immunoprecipitation (Co-IP)

According to previous studies (Cai et al., 2024; Huang et al., 2023; Jiang et al., 2019; Liu et al., 2020), to analyze the CRTC1-CREB interaction, hippocampal lysates (500 µg protein) were precleared with Protein A/G agarose beads (Santa Cruz Biotech, Santa Cruz, UNITED STATES) for 1 h at 4 °C. The supernatants were incubated overnight at 4 °C with 2 µg of anti-CRTC1 (Cell Signaling) or anti-CREB (Cell Signaling) antibody. Immune complexes were captured with Protein A/G agarose beads for 2 h at 4 °C, washed four times with lysis buffer, and eluted with 2 × Laemmli sample buffer. Co-precipitated CREB was detected by western blotting with anti-CRTC1 or anti-CREB antibody. Input samples (10% of the lysate used for IP) were run in parallel. Each sample represents an independent biological replicate from a different animal.

### 2.8 Stereotaxic surgery and viral-mediated gene knockdown

The mice were anesthetized with sodium pentobarbital (50 mg/kg, i. p.) and placed in a stereotaxic frame (RWD Life Science, Shenzhen, China). Adeno-associated virus serotype 9 (AAV9) vectors expressing short hairpin RNA (shRNA) under the U6 promoter and enhanced green fluorescent protein (EGFP) under a separate CAG promoter were used. AAV-CRTC1-shRNA and a nontargeting AAV-Control-shRNA were purchased from Genechem Co., Ltd. (Shanghai, China). Viruses (titer: 2 × 10^12^ viral genomes/ml) were bilaterally infused into the dorsal hippocampus [coordinates from bregma: AP - 2.0 mm, ML ± 1.5 mm, and DV - 1.8 mm (Cai et al., 2024; Huang et al., 2023; Jiang et al., 2019; Liu et al., 2020)] at a rate of 0.5 μl/min (1.5 µl per side) using a double-guide cannula (Plastics One, UNITED STATES) connected to a 10 µl Hamilton syringe. The needle was left in place for 5 min post-injection before withdrawal. The mice were allowed to recover for 14 days to allow maximal viral expression before further experimental procedures were performed. The sequences for control-shRNA and CRTC1-shRNA were 5′-TTC​TCC​GAA​CGT​GTC​ACG​T-3′ and 5′-GCAGTTCAA CATGATGGAGAA-3′, respectively.

### 2.9 Statistical analysis

All the data are presented as the mean ± standard error of the mean (SEM). Statistical analysis was performed using GraphPad Prism 9.0. The normality of the data distribution was assessed using the Shapiro-Wilk test. Before ANOVA, homogeneity of variance test was performed by Levene’s test. Given the relatively small sample size for molecular assays (n = 5 per group), we acknowledge that the Shapiro-Wilk test has limited power to detect deviations from normality. Therefore, we additionally performed visual inspection of Q-Q plots and residual plots to confirm approximate normality and homogeneity of variance. No significant violations of these assumptions were observed. All collected data were subjected to statistical analysis, with no data excluded. For experiments involving two independent variables (e.g., stress and drug), the data were analyzed by two-way analysis of variance (ANOVA) followed by Bonferroni’s *post hoc* correction for multiple comparisons. For viral knockdown experiments, either two-way ANOVA (drug × virus) or one-way ANOVA (followed by Tukey’s test) was employed. Statistical significance was set at *p* < 0.05. The exact F-values, degrees of freedom, and p-values are reported in the text.

### 2.10 Additional materials and methods

See the Supplementary Material for description of chronic unpredictable mild stress (CUMS), chronic restraint stress (CRS), forced swim test (FST), tail suspension test (TST), sucrose preference test (SPT), and social interaction test.

## 3 Results

### 3.1 Vortioxetine dose-dependently reverses depressive-like behaviors in male mice and chronic stress-induced alterations in hippocampal SIK2 and CRTC1 expression

**FIGURE 1:**
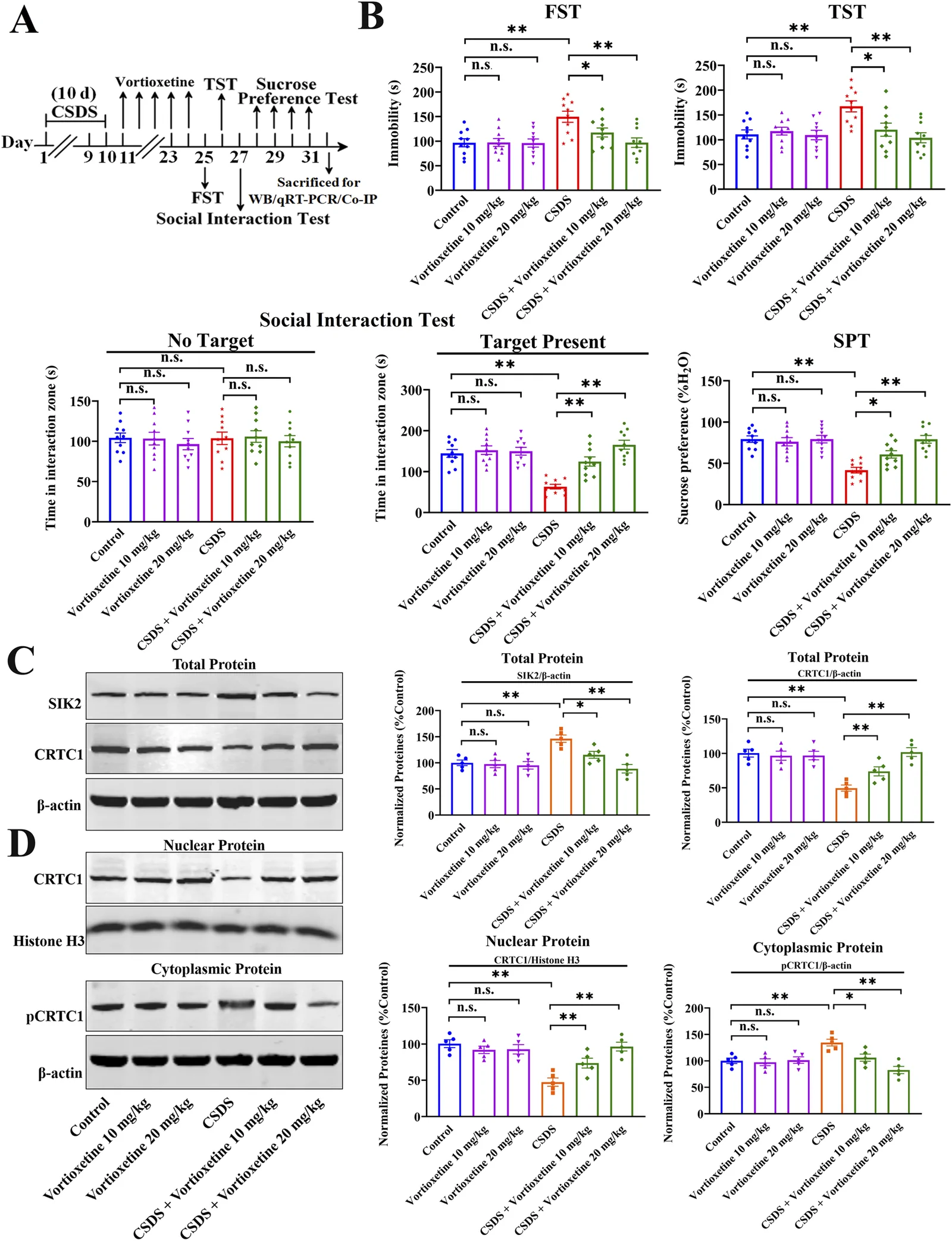
Vortioxetine treatment reverses CSDS-induced depressive-like behaviors and hippocampal SIK2-CRTC1 dysregulation in male mice. **(A)** Experimental timeline for the CSDS paradigm and vortioxetine treatment. **(B)** Vortioxetine administration (10 or 20 mg/kg, i.p.) for 2 weeks significantly ameliorated CSDS-induced behavioral deficits in male mice, as assessed by the FST, TST, sucrose preference test, and social interaction test together (n = 10). **(C)** Representative western blots and quantitative analysis showing that CSDS exposure upregulated SIK2 and downregulated total CRTC1 protein levels in the hippocampus of male mice. Vortioxetine, particularly at 20 mg/kg, dose-dependently reversed these alterations (n = 5). **(D)** Representative western blots and quantitative analysis of nuclear/cytoplasmic fractions. CSDS exposure decreased nuclear CRTC1 and increased cytoplasmic pCRTC1 protein levels in the hippocampus of male mice, whereas vortioxetine treatment restored these biological changes (n = 5). Data are presented as mean ± S.E.M. (Two-way ANOVA with Bonferroni’s *post hoc* test); *p < 0.05, **p < 0.01, n. s., not significant.

The antidepressant efficacy of vortioxetine was evaluated in the CSDS, CUMS, and CRS models. As depicted in Figures 1A,B, a two-week treatment with vortioxetine significantly reversed CSDS-induced behavioral deficits in male mice (n = 10, *p* < 0.01). Specifically, both doses (10 and 20 mg/kg) notably reduced immobility duration of CSDS-exposed mice in the FST [ANOVA: Stress F (1, 54) = 29.471, *p* < 0.0001; Drug F (2, 54) = 24.193, *p* < 0.0001; Interaction F (2, 54) = 18.244, *p* < 0.0001] and TST [ANOVA: Stress F (1, 54) = 25.888, *p* < 0.0001; Drug F (2, 54) = 20.156, *p* < 0.0001; Interaction F (2, 54) = 15.677, *p* < 0.0001], evidently restored sucrose preference of CSDS-exposed mice [ANOVA: Stress F (1, 54) = 31.557, *p* < 0.0001; Drug F (2, 54) = 26.842, *p* < 0.0001; Interaction F (2, 54) = 21.095, *p* < 0.0001], and remarkably increased social interaction of CSDS-exposed mice [ANOVA: Stress F (1, 54) = 48.225, *p* < 0.0001; Drug F (2, 54) = 35.114, *p* < 0.0001; Interaction F (2, 54) = 28.463, *p* < 0.0001]. The 20 mg/kg dose demonstrated superior efficacy across all behavioral measures, reaching statistical equivalence to healthy control levels.

**FIGURE 2:**
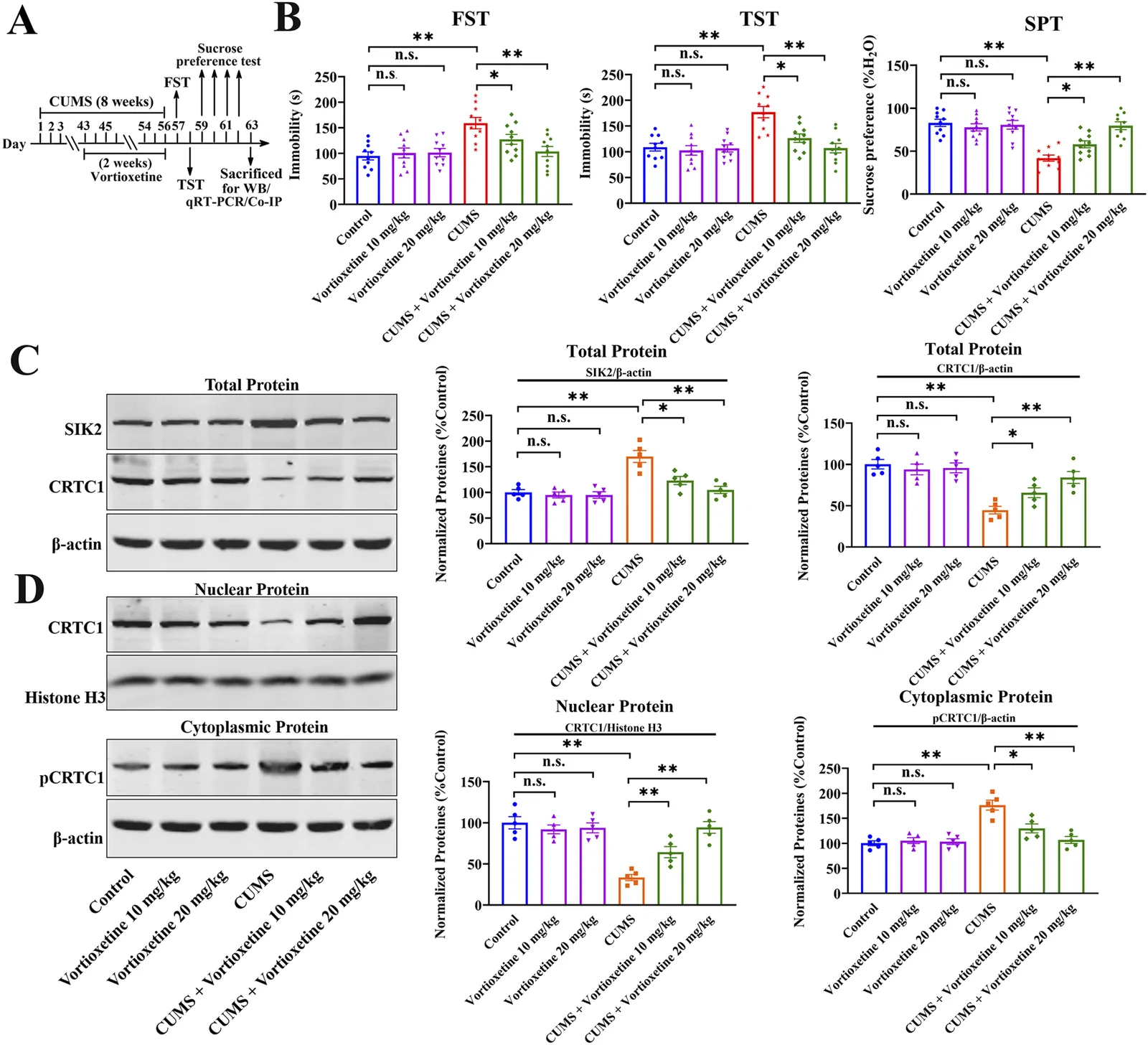
Vortioxetine treatment attenuates CUMS-induced depressive-like behaviors and hippocampal SIK2-CRTC1 dysregulation in male mice. **(A)** Experimental timeline for the CUMS paradigm and vortioxetine treatment. **(B)** CUMS exposure increased immobility time (FST, TST) and decreased sucrose preference in male mice, all of which were dose-dependently reversed by vortioxetine treatment (10 or 20 mg/kg, i.p.) during the final 2 weeks (n = 10). **(C)** Representative western blots and quantitative analysis showing CUMS-induced upregulation of hippocampal SIK2 and downregulation of total CRTC1 in male mice, which were counteracted by vortioxetine treatment (n = 5). **(D)** Representative western blots and quantitative analysis demonstrating that CUMS exposure reduced nuclear CRTC1 and increased cytoplasmic pCRTC1 in the hippocampus of male mice, effects that were reversed by vortioxetine administration (n = 5). Data are presented as mean ± S.E.M. (Two-way ANOVA with Bonferroni’s *post hoc* test); *p < 0.05, **p < 0.01, n. s., not significant.

Similarly, vortioxetine treatment fully alleviated CUMS-induced depressive-like phenotypes in male mice (Figures 2A,B; n = 10, *p* < 0.01). Administration of vortioxetine significantly decreased immobility duration of CUMS-exposed mice in the FST [ANOVA: Stress F (1, 54) = 27.196, *p* < 0.0001; Drug F (2, 54) = 22.735, *p* < 0.0001; Interaction F (2, 54) = 17.128, *p* < 0.0001] and TST [ANOVA: Stress F (1, 54) = 32.449, *p* < 0.0001; Drug F (2, 54) = 27.918, *p* < 0.0001; Interaction F (2, 54) = 22.437, *p* < 0.0001], and enhanced sucrose preference of CUMS-exposed mice [ANOVA: Stress F (1, 54) = 26.838, *p* < 0.0001; Drug F (2, 54) = 23.965, *p* < 0.0001; Interaction F (2, 54) = 19.211, *p =*, *p* < 0.0001] in a dose-dependent manner.

**FIGURE 3:**
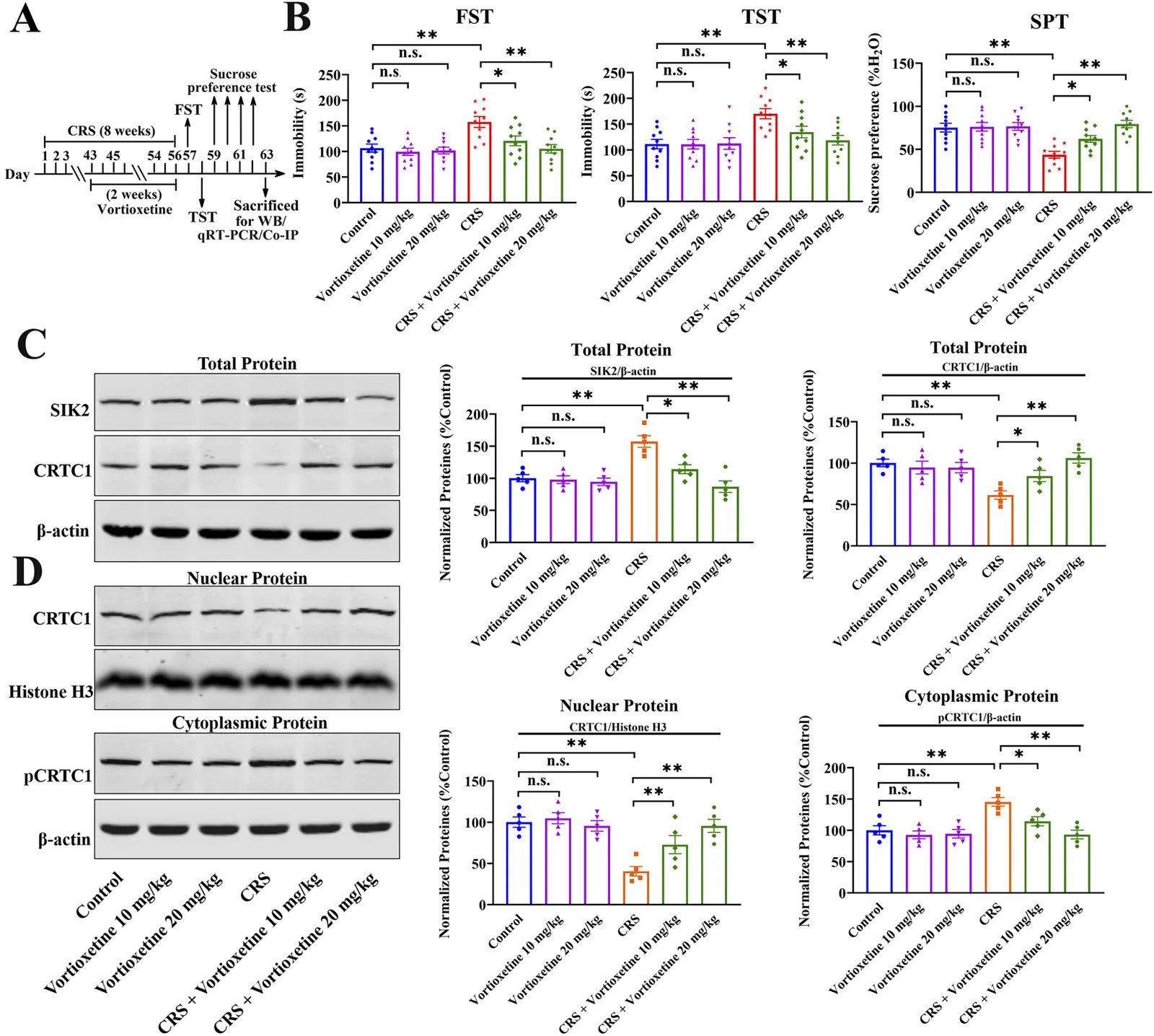
Vortioxetine treatment attenuates CRS-induced depressive-like behaviors and hippocampal SIK2-CRTC1 dysregulation in male mice. **(A)** Experimental timeline for the CRS paradigm and vortioxetine treatment. **(B)** CRS exposure increased immobility time (FST, TST) and decreased sucrose preference in male mice, all of which were dose-dependently reversed by vortioxetine treatment (10 or 20 mg/kg, i.p.) during the final 2 weeks (n = 10). **(C)** Representative western blots and quantitative analysis showing CRS-induced upregulation of hippocampal SIK2 and downregulation of total CRTC1 in male mice, which were counteracted by vortioxetine treatment (n = 5). **(D)** Representative western blots and quantitative analysis demonstrating that CRS exposure reduced nuclear CRTC1 and increased cytoplasmic pCRTC1 in the hippocampus of male mice, effects that were reversed by vortioxetine administration (n = 5). Data are presented as mean ± S.E.M. (Two-way ANOVA with Bonferroni’s *post hoc* test); *p < 0.05, **p < 0.01, n. s., not significant.

Moreover, vortioxetine treatment evidently ameliorated CRS-induced depressive-like phenotypes in male mice (Figures 3A,B; n = 10, *p* < 0.01). Administration of vortioxetine notably decreased immobility duration of CRS-exposed mice in the FST [ANOVA: Stress F (1, 54) = 25.187, *p* < 0.0001; Drug F (2, 54) = 20.841, *p* < 0.0001; Interaction F (2, 54) = 15.269, *p* < 0.0001] and TST [ANOVA: Stress F (1, 54) = 30.594, *p* < 0.0001; Drug F (2, 54) = 24.862, *p* < 0.0001; Interaction F (2, 54) = 19.075, *p* < 0.0001], and enhanced sucrose preference of CRS-exposed mice [ANOVA: Stress F (1, 54) = 28.161, *p* < 0.0001; Drug F (2, 54) = 22.994, *p* < 0.0001; Interaction F (2, 54) = 16.285, *p* < 0.0001] in a dose-dependent manner.

Subsequent molecular analyses revealed that CSDS, CUMS, and CRS all robustly upregulated hippocampal SIK2 protein expression while downregulating protein expression of total CRTC1 in male mice (Figures 1C, 2C, 3C; n = 5, *p* < 0.01). Vortioxetine treatment, particularly at 20 mg/kg, dose-dependently reversed these stress-induced alterations (Figures 1C, 2C, 3C; n = 5, *p* < 0.01). For CSDS: SIK2 [ANOVA: Stress F (1, 24) = 36.772, *p* < 0.0001; Drug F (2, 24) = 29.441, *p* < 0.0001; Interaction F (2, 24) = 23.102, *p* < 0.0001], total CRTC1 [ANOVA: Stress F (1, 24) = 28.159, *p* < 0.0001; Drug F (2, 24) = 22.115, *p* < 0.0001; Interaction F (2, 24) = 17.334, *p* < 0.0001]. For CUMS: SIK2 [ANOVA: Stress F (1, 24) = 42.955, *p* < 0.0001; Drug F (2, 24) = 34.688, *p* < 0.0001; Interaction F (2, 24) = 26.704, *p* < 0.0001], total CRTC1 [ANOVA: Stress F (1, 24) = 30.574, *p* < 0.0001; Drug F (2, 24) = 25.048, *p* < 0.0001; Interaction F (2, 24) = 19.231, *p* < 0.0001]. For CRS: SIK2 [ANOVA: Stress F (1, 24) = 36.187, *p* < 0.0001; Drug F (2, 24) = 31.705, *p* < 0.0001; Interaction F (2, 24) = 23.664, *p* < 0.0001], total CRTC1 [ANOVA: Stress F (1, 24) = 27.156, *p* < 0.0001; Drug F (2, 24) = 22.806, *p* < 0.0001; Interaction F (2, 24) = 17.033, *p* < 0.0001]. Vortioxetine treatment had no effect on these markers in non-stressed control mice (Figures 1C, 2C, 3C; n = 5).

**FIGURE 4:**
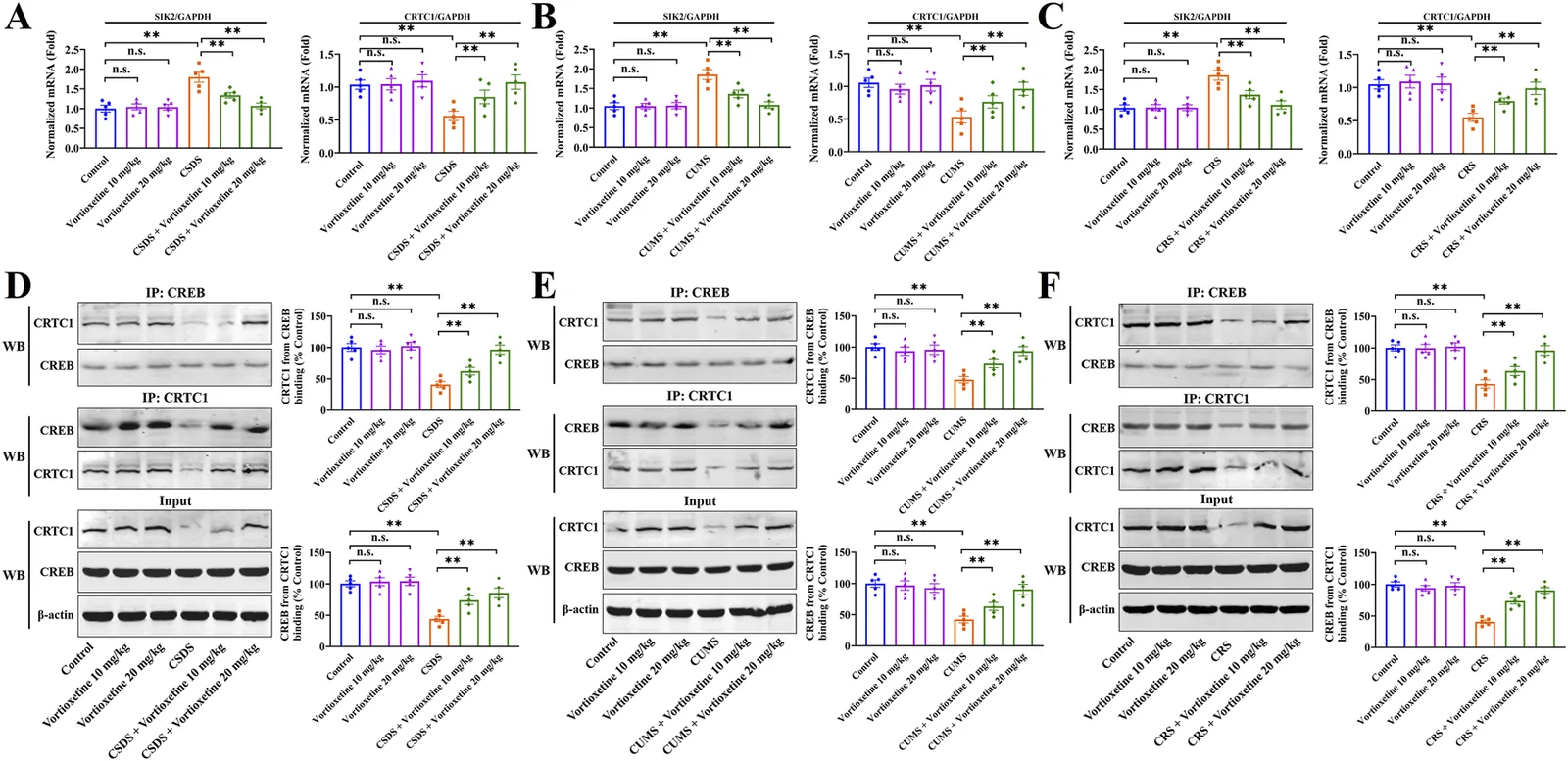
Vortioxetine counteracts chronic stress-induced alterations in hippocampal SIK2/CRTC1 mRNA and CRTC1-CREB binding in male mice. **(A–C)** qRT-PCR analysis of hippocampal SIK2 and CRTC1 mRNA levels. CSDS **(A)** CUMS **(B)** and CRS **(C)** all significantly increased SIK2 mRNA and decreased CRTC1 mRNA in the hippocampus of male mice, and these transcriptional changes were dose-dependently reversed by vortioxetine treatment (n = 5). **(D–F)** Co-IP analysis of CRTC1-CREB interaction in hippocampal extracts. Representative western blots and quantitative analysis show that CSDS **(D)** CUMS **(E)** and CRS **(F)** all impaired the physical association between CRTC1 and CREB in the hippocampus of male mice, which was significantly restored by vortioxetine administration (10 or 20 mg/kg) (n = 5). Data are presented as mean ± S.E.M. (Two-way ANOVA with Bonferroni’s *post hoc* test); **p < 0.01, n. s., not significant.

We further examined the mRNA levels of SIK2 and CRTC1 in the hippocampus of male mice using qRT-PCR. Consistent with the protein data, Figure 4A shows that compared to the control group, CSDS exposure significantly increased hippocampal SIK2 mRNA level [ANOVA: Stress F (1, 24) = 39.741, *p* < 0.0001; Drug F (2, 24) = 31.883, *p* < 0.0001; Interaction F (2, 24) = 25.106, *p* < 0.0001] and decreased CRTC1 mRNA level [ANOVA: Stress F (1, 24) = 30.625, *p* < 0.0001; Drug F (2, 24) = 24.192, *p* < 0.0001; Interaction F (2, 24) = 19.384, *p* < 0.0001] (n = 5, *p* < 0.01). Vortioxetine treatment, especially at 20 mg/kg, dose-dependently reversed these changes (n = 5, *p* < 0.01). Similarly, Figure 4B indicates that CUMS exposure markedly upregulated hippocampal SIK2 mRNA [ANOVA: Stress F (1, 24) = 45.225, *p* < 0.0001; Drug F (2, 24) = 36.774, *p* < 0.0001; Interaction F (2, 24) = 29.855, *p* < 0.0001] and downregulated CRTC1 mRNA [ANOVA: Stress F (1, 24) = 33.419, *p* < 0.0001; Drug F (2, 24) = 27.558, *p* < 0.0001; Interaction F (2, 24) = 21.774, *p* < 0.0001], which were effectively antagonized by vortioxetine treatment (n = 5, *p* < 0.01). Moreover, Figure 4C reveals that CRS exposure also evidently upregulated hippocampal SIK2 mRNA [ANOVA: Stress F (1, 24) = 40.691, *p* < 0.0001; Drug F (2, 24) = 33.776, *p* < 0.0001; Interaction F (2, 24) = 25.421, *p* < 0.0001] and downregulated CRTC1 mRNA [ANOVA: Stress F (1, 24) = 30.199, *p* < 0.0001; Drug F (2, 24) = 25.442, *p* < 0.0001; Interaction F (2, 24) = 18.375, *p* < 0.0001], which were significantly restored by vortioxetine treatment (n = 5, *p* < 0.01). Vortioxe0tine administration did not affect hippocampal SIK2 and CRTC1 mRNA levels in naïve control mice (Figures 4A–C; n = 5).

### 3.2 Vortioxetine restores chronic stress-impaired CRTC1 nuclear translocation and CRTC1-CREB binding in the hippocampus of male mice

We next examined the impact of vortioxetine on the subcellular localization of CRTC1. Western blot analysis of nuclear and cytoplasmic fractions showed that chronic stress (CSDS, CUMS, and CRS) significantly decreased nuclear CRTC1 and increased cytoplasmic phosphorylated CRTC1 (pCRTC1) levels in the hippocampus of male mice (Figures 1D, 2D, 3D; n = 5, *p* < 0.01). Both doses of vortioxetine reversed these changes, with the higher dose (20 mg/kg) showing a more complete restoration to control levels (Figures 1D, 2D, 3D; n = 5, *p* < 0.01). For CSDS: nuclear CRTC1 [ANOVA: Stress F (1, 24) = 34.552, *p* < 0.0001; Drug F (2, 24) = 27.629, *p* < 0.0001; Interaction F (2, 24) = 21.887, *p* < 0.0001]; cytoplasmic pCRTC1 [ANOVA: Stress F (1, 24) = 29.882, *p* < 0.0001; Drug F (2, 24) = 24.206, *p* < 0.0001; Interaction F (2, 24) = 19.441, *p* < 0.0001]. For CUMS: nuclear CRTC1 [ANOVA: Stress F (1, 24) = 46.577, *p* < 0.0001; Drug F (2, 24) = 36.718, *p* < 0.0001; Interaction F (2, 24) = 28.511, *p* < 0.0001]; cytoplasmic pCRTC1 [ANOVA: Stress F (1, 24) = 38.265, *p* < 0.0001; Drug F (2, 24) = 31.449, *p* < 0.0001; Interaction F (2, 24) = 24.774, *p* < 0.0001]. For CRS: nuclear CRTC1 [ANOVA: Stress F (1, 24) = 41.628, *p* < 0.0001; Drug F (2, 24) = 32.051, *p* < 0.0001; Interaction F (2, 24) = 26.294, *p* < 0.0001]; cytoplasmic pCRTC1 [ANOVA: Stress F (1, 24) = 35.101, *p* < 0.0001; Drug F (2, 24) = 29.748, *p* < 0.0001; Interaction F (2, 24) = 22.396, *p* < 0.0001].

To determine the functional consequence of restored nuclear CRTC1, we assessed CRTC1-CREB interaction via Co-IP. As shown in Figures 4D–F, chronic stress (CSDS, CUMS, and CRS) markedly reduced the binding between CRTC1 and CREB in hippocampal nuclear extracts of male mice (n = 5, *p* < 0.01). Vortioxetine treatment (10 and 20 mg/kg) significantly recovered this interaction (Figures 4D–F; n = 5, *p* < 0.01). Specifically, the Co-IP results in the reciprocal direction (immunoprecipitating CREB and blotting for CRTC1; immunoprecipitating CRTC1 and blotting for CREB) were completely consistent. For CSDS: CRTC1 from CREB binding [ANOVA: Stress F (1, 24) = 33.924, *p* < 0.0001; Drug F (2, 24) = 26.088, *p* < 0.0001; Interaction F (2, 24) = 20.763, *p* < 0.0001]; CREB from CRTC1 binding [ANOVA: Stress F (1, 24) = 32.529, *p* < 0.0001; Drug F (2, 24) = 24.661, *p* < 0.0001; Interaction F (2, 24) = 18.772, *p* < 0.0001]. For CUMS: CRTC1 from CREB binding [ANOVA: Stress F (1, 24) = 37.569, *p* < 0.0001; Drug F (2, 24) = 25.764, *p* < 0.0001; Interaction F (2, 24) = 19.031, *p* < 0.0001]; CREB from CRTC1 binding [ANOVA: Stress F (1, 24) = 31.609, *p* < 0.0001; Drug F (2, 24) = 23.995, *p* < 0.0001; Interaction F (2, 24) = 17.184, *p* < 0.0001]. For CRS: CRTC1 from CREB binding [ANOVA: Stress F (1, 24) = 30.217, *p* < 0.0001; Drug F (2, 24) = 25.303, *p* < 0.0001; Interaction F (2, 24) = 16.699, *p* < 0.0001]; CREB from CRTC1 binding [ANOVA: Stress F (1, 24) = 28.769, *p* < 0.0001; Drug F (2, 24) = 22.113, *p* < 0.0001; Interaction F (2, 24) = 15.465, *p* < 0.0001].

### 3.3 Hippocampal CRTC1 is required for the antidepressant effects of vortioxetine

**FIGURE 5:**
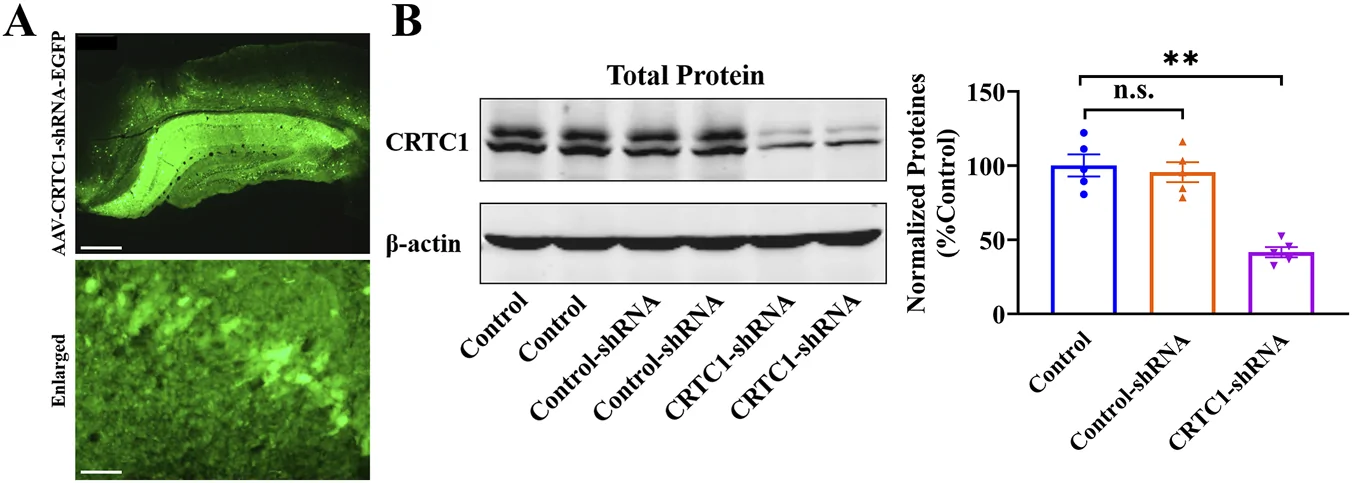
Validation of efficient hippocampal CRTC1 knockdown via AAV-shRNA. **(A)** Representative fluorescence microscopy images (left) showing EGFP expression in the hippocampus of male mice 2 weeks after stereotactic infusion of AAV-CRTC1-shRNA-EGFP. Scale bars: 200 µm (overview), 25 µm (enlarged). **(B)** Western blot analysis (right) confirming a significant reduction in hippocampal CRTC1 protein level in male mice infused with CRTC1-shRNA compared to those infused with Control-shRNA or non-infused controls (n = 5). Data are presented as mean ± S.E.M. (One-way ANOVA with Tukey’s *post hoc* test); **p < 0.01, n. s., not significant.

To establish a causal role for hippocampal CRTC1 in vortioxetine’s action, we employed AAV-mediated CRTC1 knockdown. The efficacy of AAV-CRTC1-shRNA in reducing hippocampal CRTC1 expression in male mice by approximately 60% was confirmed (Figures 5A,B, n = 5, *p* < 0.01; ANOVA: F (2, 12) = 41.283, *p* < 0.0001).

**FIGURE 6:**
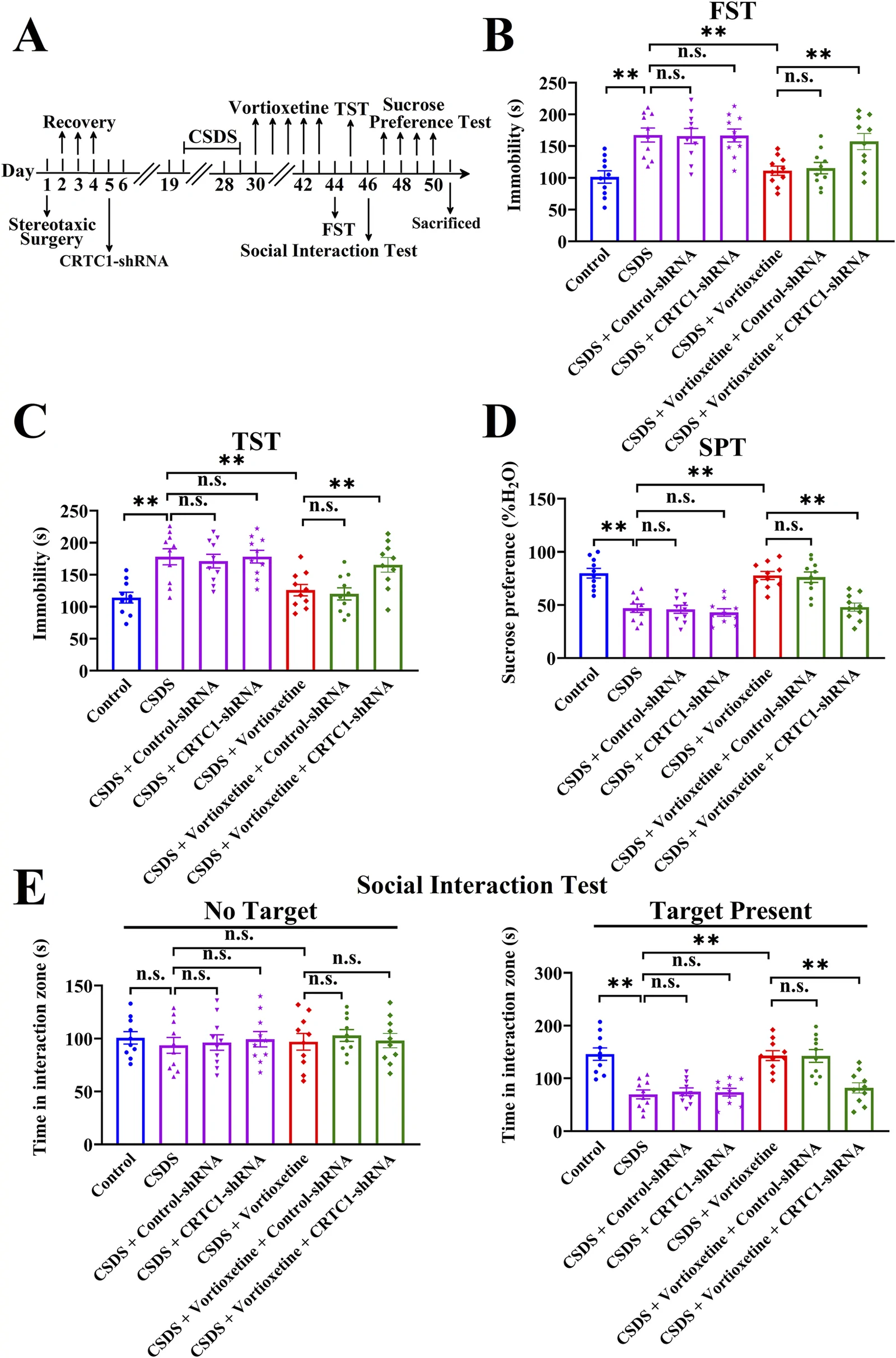
Hippocampal CRTC1 knockdown abolishes the antidepressant effects of vortioxetine in the CSDS model of male mice. **(A)** Experimental timeline combining hippocampal CRTC1 knockdown, CSDS exposure, and vortioxetine (20 mg/kg) treatment. **(B–E)** Behavioral results showing that the therapeutic effects of vortioxetine on FST immobility **(B)** TST immobility **(C)** sucrose preference **(D)** and social interaction **(E)** in CSDS-exposed mice were completely blocked in mice with hippocampal CRTC1 knockdown (CRTC1-shRNA), but not in those infused with Control-shRNA (n = 10). Data are presented as mean ± S.E.M. (Two-way ANOVA with Bonferroni’s *post hoc* test); **p < 0.01, n. s., not significant.

**FIGURE 7:**
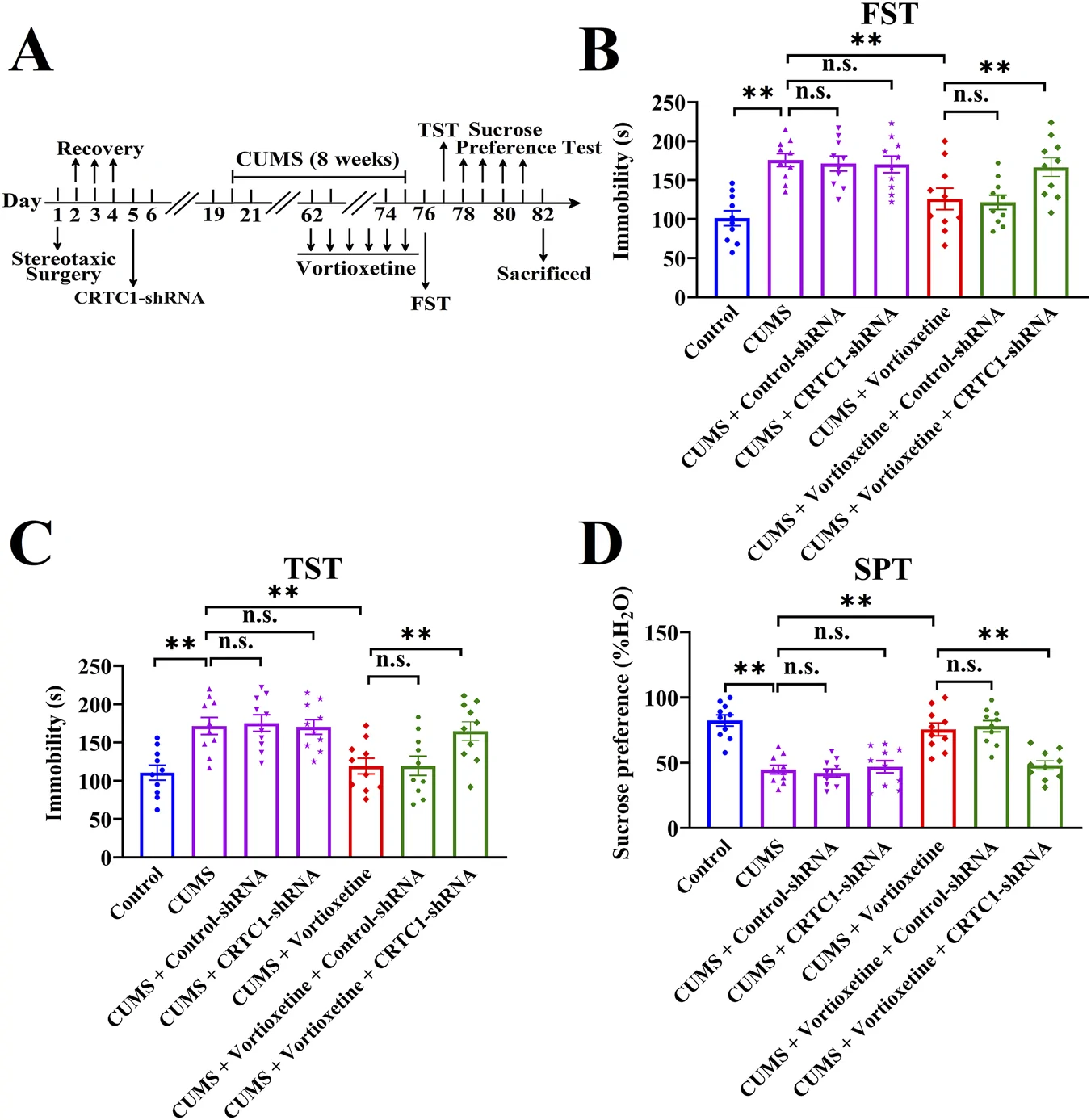
Hippocampal CRTC1 is necessary for the antidepressant efficacy of vortioxetine in the CUMS model of male mice. **(A)** Experimental timeline combining hippocampal CRTC1 knockdown, CUMS exposure, and vortioxetine (20 mg/kg) treatment. **(B–D)** The behavioral improvements induced by vortioxetine in CUMS-exposed mice—including reduced immobility in the FST **(B)** and TST **(C)** and restored sucrose preference **(D)** were absent in mice with hippocampal CRTC1 knockdown (CRTC1-shRNA). The usage of Control-shRNA did not affect vortioxetine’s efficacy (n = 10). Data are presented as mean ± S.E.M. (Two-way ANOVA with Bonferroni’s *post hoc* test); **p < 0.01, n. s., not significant.

**FIGURE 8:**
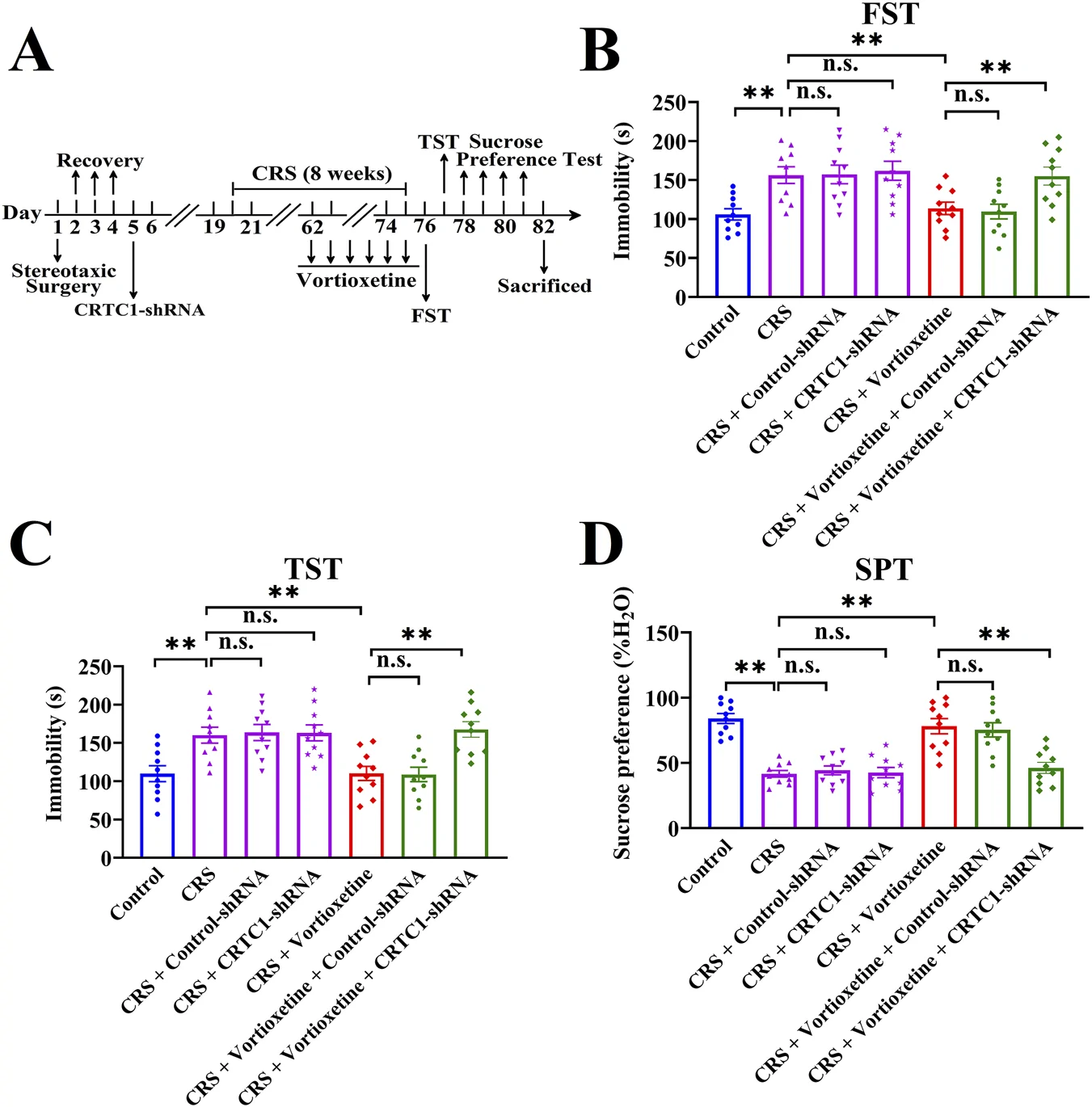
Hippocampal CRTC1 is required for the antidepressant actions of vortioxetine in the CRS model of male mice. **(A)** Experimental timeline combining hippocampal CRTC1 knockdown, CRS exposure, and vortioxetine (20 mg/kg) treatment. **(B–D)** The behavioral improvements induced by vortioxetine in CRS-exposed mice—including reduced immobility in the FST **(B)** and TST **(C)** and restored sucrose preference **(D)** were absent in mice with hippocampal CRTC1 knockdown (CRTC1-shRNA). The usage of Control-shRNA did not affect vortioxetine’s efficacy (n = 10). Data are presented as mean ± S.E.M. (Two-way ANOVA with Bonferroni’s *post hoc* test); **p < 0.01, n. s., not significant.

In CSDS-exposed mice, the antidepressant effects of vortioxetine (20 mg/kg) were completely abolished by prior hippocampal CRTC1 knockdown (Figures 6A–E). Mice in the (Vortioxetine + CRTC1-shRNA + CSDS)-treated group displayed significantly longer immobility in the FST [ANOVA: shRNA, F (1, 36) = 21.517, *p* < 0.0001; Drug, F (1, 36) = 26.026, *p* < 0.0001; Interaction, F (1, 36) = 18.069, *p* < 0.0001] and TST [ANOVA: shRNA, F (1, 36) = 20.954, *p* < 0.0001; Drug, F (1, 36) = 25.152, *p* < 0.0001; Interaction, F (1, 36) = 16.239, *p* < 0.0001], lower sucrose preference [ANOVA: shRNA, F (1, 36) = 17.506, *p* < 0.0001; Drug, F (1, 36) = 20.669, *p* < 0.0001; Interaction, F (1, 36) = 14.328, *p* < 0.0001], and reduced social interaction [ANOVA: shRNA, F (1, 36) = 27.144, *p* < 0.0001; Drug, F (1, 36) = 33.609, *p* < 0.0001; Interaction, F (1, 36) = 22.501, *p* < 0.0001] compared to mice in both the (Vortioxetine + CSDS)-treated and (Vortioxetine + Control-shRNA + CSDS)-treated groups (n = 10, *p* < 0.01). Similarly, in the CUMS model, CRTC1 knockdown notably blocked the therapeutic efficacy of vortioxetine in male mice (Figures 7A–D). Mice in the (Vortioxetine + CRTC1-shRNA + CUMS)-treated group showed evidently more FST immobility [ANOVA: shRNA, F (1, 36) = 24.302, *p* < 0.0001; Drug, F (1, 36) = 28.099, *p* < 0.0001; Interaction, F (1, 36) = 19.184, *p* < 0.0001], more TST immobility [ANOVA: shRNA, F (1, 36) = 24.093, *p* < 0.0001; Drug, F (1, 36) = 27.662, *p* < 0.0001; Interaction, F (1, 36) = 18.578, *p* < 0.0001], and less sucrose preference [ANOVA: shRNA, F (1, 36) = 20.724, *p* < 0.0001; Drug, F (1, 36) = 24.339, *p* < 0.0001; Interaction, F (1, 36) = 15.912, *p* < 0.0001] than mice in both the (Vortioxetine + CUMS)-treated and (Vortioxetine + Control-shRNA + CUMS)-treated groups (n = 10, *p* < 0.01). Moreover, in the CRS model, CRTC1 knockdown also significantly attenuated the therapeutic actions of vortioxetine in male mice (Figures 8A–D). Mice in the (Vortioxetine + CRTC1-shRNA + CRS)-treated group exhibited obviously more FST immobility [ANOVA: shRNA, F (1, 36) = 19.002, *p* < 0.0001; Drug, F (1, 36) = 23.982, *p* < 0.0001; Interaction, F (1, 36) = 15.195, *p* < 0.0001], more TST immobility [ANOVA: shRNA, F (1, 36) = 21.994, *p* < 0.0001; Drug, F (1, 36) = 26.817, *p* < 0.0001; Interaction, F (1, 36) = 17.045, *p* < 0.0001], and less sucrose preference [ANOVA: shRNA, F (1, 36) = 16.403, *p* < 0.0001; Drug, F (1, 36) = 22.593, *p* < 0.0001; Interaction, F (1, 36) = 13.805, *p* < 0.0001] than mice in both the (Vortioxetine + CRS)-treated and (Vortioxetine + Control-shRNA + CRS)-treated groups (n = 10, *p* < 0.01). The usage of Control-shRNA had no effects (n = 10).

Taken together, these results demonstrate that the multimodal antidepressant vortioxetine effectively ameliorates chronic stress-induced depressive-like behaviors and concomitant dysregulation of the hippocampal SIK2-CRTC1-CREB axis in male mice in a dose-dependent manner, and that the functional integrity of hippocampal CRTC1 is essential for its therapeutic action.

## 4 Discussion

While the serotonergic system remains a cornerstone of antidepressant pharmacology, the delayed therapeutic onset and variable efficacy of conventional monoaminergic agents underscore the complexity of MDD and necessitate exploration of downstream signaling mechanisms (Boku et al., 2018; Gonda et al., 2023). The present study provides novel evidence that vortioxetine, a multimodal antidepressant with a distinct receptor profile, exerts its therapeutic effects in male mice through modulation of hippocampal SIK2 and CRTC1. Our findings demonstrate that chronic stress-induced upregulation of hippocampal SIK2 and subsequent impairment of CRTC1 function are critical pathological features in three well-validated depression models in male mice, and that vortioxetine dose-dependently reverses these molecular deficits while restoring behavioral homeostasis. Crucially, we establish that intact hippocampal CRTC1 signaling is essential for vortioxetine’s antidepressant efficacy in male mice, providing mechanistic insight into its unique therapeutic profile.

The multimodal pharmacology of vortioxetine—combining serotonin reuptake inhibition with direct modulation of several 5-HT receptor subtypes—distinguishes it from conventional SSRIs (Sanchez et al., 2015). This study reveals that beyond enhancing serotonergic tone, vortioxetine engages fundamental plasticity-regulating pathways in the hippocampus. The dose-dependent normalization of both protein and mRNA levels of SIK2 and CRTC1 suggests that vortioxetine acts at transcriptional and/or post-transcriptional levels to rebalance this key signaling axis. Notably, the superior efficacy of the higher dose (20 mg/kg) across both behavioral and molecular endpoints mirrors clinical observations where vortioxetine exhibits dose-dependent therapeutic effects, particularly on cognitive symptoms often refractory to SSRIs (Bennabi et al., 2019; Huang et al., 2022; Sanchez et al., 2015). This correlation strengthens the translational relevance of our findings.

The molecular cascade elucidated here—from SIK2 downregulation to CRTC1 nuclear translocation and enhanced CREB binding—provides a plausible explanation for vortioxetine’s documented pro-cognitive and neuroplastic effects (Bennabi et al., 2019; Huang et al., 2022; Waller et al., 2017b). Previous studies have shown that vortioxetine enhances hippocampal synaptic plasticity, increases expression of plasticity-related genes, and promotes neurogenesis (Bétry et al., 2015; Dale et al., 2014; Guilloux et al., 2013; Waller et al., 2017a; Waller et al., 2017b). Our results position hippocampal SIK2 and CRTC1 as possible molecules mediating these effects. The restoration of CRTC1-CREB interaction by vortioxetine is particularly significant, as this transcriptional complex regulates numerous genes involved in synaptic assembly, neuronal differentiation, and metabolic adaptation—processes increasingly implicated in depression pathophysiology.

Interestingly, unlike some conventional antidepressants (fluoxetine, venlafaxine, and mirtazapine) that affect hippocampal SIK2 and CRTC1 under basal conditions (Jiang et al., 2019), vortioxetine’s modulatory effects in our study were primarily observed in stress-exposed animals. This “pathology-dependent” action suggests a targeted normalization of dysregulated signaling rather than broad pharmacological perturbation, potentially correlating with vortioxetine’s favorable tolerability profile in clinical use (Sowa-Kućma et al., 2017). The precise upstream mechanisms through which vortioxetine’s complex receptor pharmacology converges on SIK2 regulation warrant further investigation. Potential mediators include the 5-HT_1A_ receptor (linked to cAMP/PKA signaling), 5-HT_1B_ auto-receptors regulating serotonin release dynamics, and 5-HT_3_ receptor antagonism modulating inter-neuronal activity and downstream calcium signaling—all of which could influence SIK2 activity and CRTC1 phosphorylation status (Henriksson et al., 2012; Lee et al., 2014; Ricarte et al., 2018; Sasaki et al., 2011).

Our knockdown experiments provide definitive causal evidence that hippocampal CRTC1 is not merely correlated with but is necessary for vortioxetine’s behavioral efficacy in male mice. The complete abolition of therapeutic effects in CRTC1-deficient animals across multiple behavioral domains (despair, anhedonia, social avoidance) underscores the centrality of this molecule. This finding has important implications for treatment stratification, suggesting that patients with pronounced hippocampal CRTC1 dysfunction might particularly benefit from vortioxetine treatment. Future clinical studies examining CRTC1-related biomarkers could test this hypothesis.

Several study limitations should be acknowledged. First, while CSDS, CUMS, and CRS model specific depression-related dimensions, they cannot fully capture the heterogeneity of human MDD. Second, our exclusive focus on male mice necessitates future research in females to assess potential sex differences in pathway engagement. Third, the specific 5-HT receptor subtypes mediating vortioxetine’s effects on SIK2-CRTC1 signaling remain to be dissected using selective pharmacological tools. Fourth, due to limited funds and resources, we did not include independent locomotor control measures (e.g., open-field test). Thus, we cannot completely exclude the possibility that nonspecific effects on general activity or arousal contributed to the observed behavioral readouts, and future studies incorporating locomotor control measures are warranted. Fifth, for molecular analyses (western blotting, qRT-PCR, subcellular fractionation, and Co-IP), we used a sample size of n = 5 per group. While this sample size is consistent with common practice in the field, we recognize that it is relatively small, which may increase sensitivity to outliers and reduce the reliability of normality tests such as the Shapiro-Wilk test. Although we supplemented normality assessment with visual inspection of Q-Q plots and residual plots to mitigate this concern, we cannot completely exclude the possibility that undetected non-normality or variance instability may have influenced the statistical inferences. Future studies with larger sample sizes would provide more robust confirmation of these molecular findings. Finally, while we demonstrate CRTC1 necessity through knockdown approaches, complementary rescue experiments would further solidify the mechanistic chain. Despite these limitations, our findings significantly advance understanding of vortioxetine’s mechanism of action. By bridging its unique receptor pharmacology with fundamental neuroplasticity pathways, we provide a molecular framework explaining its clinical advantages over conventional antidepressants. Furthermore, this study reinforces hippocampal SIK2 and CRTC1 as feasible therapeutic targets for mood disorders. The convergence of multiple antidepressants—now including both conventional SSRIs/SNRIs (fluoxetine, paroxetine, and venlafaxine) and multimodal agents like vortioxetine—on the two molecules suggest their fundamental role in depression pathophysiology and treatment response. Future development of direct SIK2 modulators or CRTC1 activators, guided by the mechanistic insights from antidepressant studies, may yield novel therapeutics with improved efficacy and onset profiles.

We acknowledge that hippocampal SIK2 and CRTC1 have been previously linked to conventional monoamine-based antidepressants (SSRIs/SNRIs). However, whether a multimodal antidepressant with a complex receptor pharmacology (5-HT transporter inhibition combined with multiple 5-HT receptor modulations) converges on the same downstream molecules remained unknown. The incremental contribution of this study is to demonstrate that despite vortioxetine’s distinct upstream mechanism of action, its antidepressant effects ultimately converge on the same molecules as simpler reuptake inhibitors. This finding strengthens the generalizability and centrality for hippocampal SIK2 and CRTC1 as common targets for chemically and pharmacologically diverse antidepressants, rather than being specific to a single drug class.

In conclusion, this study positions vortioxetine not merely as a serotonergic modulator but as a targeted regulator of stress-induced hippocampal plasticity deficits. By demonstrating its dose-dependent normalization of hippocampal SIK2/CRTC1-related signaling and establishing CRTC1’s essential role in its therapeutic action in male mice, we provide a novel mechanistic foundation for vortioxetine’s clinical profile and contribute to the evolving understanding of depression as a disorder of impaired neuroplasticity.

## Data Availability

The raw data supporting the conclusions of this article will be made available by the authors, without undue reservation.
