# Supplementary material for: The multimodal antidepressant vortioxetine exerts therapeutic effects in male mice which is associated with normalization of hippocampal SIK2/CRTC1-related signaling and require hippocampal CRTC1
Source: Front Pharmacol. 2026 Aug 20;17:1837566. doi: 10.3389/fphar.2026.1837566 (PMC13537961; doi:10.3389/fphar.2026.1837566)
Supplement: Supplementary file 1 [file DataSheet1.docx]

**Supplemental Information**

**Materials and Methods**

*Chronic Unpredictable Mild Stress (CUMS)*

The CUMS procedure was performed according to previous studies (Cai et al., 2024; Huang et al., 2023; Jiang et al., 2019; Liu et al., 2020). Over 8 weeks, singly-housed C57BL/6J mice were exposed to two different mild stressors per day in a randomized order. The stressors included: 45° cage tilt (12 h), damp bedding (12 h), confinement in a restrained tube (1 h), continuous illumination (12 h), food/water deprivation (23 h), white noise (85 dB, 4 h), cold stress (4 °C, 1 h), cage rotation (30 min), and overnight inversion of the light/dark cycle. Non-stressed control mice were group-housed and handled daily. Drug treatments were administered once daily during the final 2 weeks of the CUMS regimen.

*Chronic Restraint Stress (CRS)*

For the CRS model, the procedure was performed according to previous studies (Tian et al., 2024; Zhao et al., 2025). C57BL/6J mice in the stressed group were individually subjected to 3 h of CRS daily (from 9:00 a.m. to 12:00 p.m.) for 8 weeks, with daily administration of drugs during the last 2 weeks. The restraint stress was conducted using 50 ml conical plastic tubes with ventilation holes. Mice in the control group received only daily handling.

*Forced Swim Test (FST)*

According to previous studies (Cai et al., 2024; Huang et al., 2023; Jiang et al., 2019; Liu et al., 2020), mice were placed individually in clear Plexiglas cylinders (25 cm height × 10 cm diameter) filled to a depth of 15 cm with water (24 ± 1 °C). A 6-minute test session was video recorded. Immobility time (defined as passive floating with only minimal movements necessary to keep the head above water) during the final 4 minutes was scored manually by an observer blinded to group assignment.

*Tail Suspension Test (TST)*

According to previous studies (Cai et al., 2024; Huang et al., 2023; Jiang et al., 2019; Liu et al., 2020), mice were suspended by the tail using adhesive tape (-2 cm from the tip) from a horizontal bar 50 cm above the floor. The 6-minute session was recorded, and the total immobility time (defined as the complete absence of limb or body movement) was scored by an observer blinded to group assignment.

*Sucrose Preference Test (SPT)*

According to previous studies (Cai et al., 2024; Huang et al., 2023; Jiang et al., 2019; Liu et al., 2020), anhedonia was assessed using a two-bottle choice paradigm. After 48 hours of acclimatization to 1% sucrose solution, the mice were deprived of food and water for 18 hours. They were then presented with two preweighed bottles, one containing 1% sucrose and the other containing tap water, for a 6-hour period. Bottle positions were switched at the 3-hour mark to control for side preference. Sucrose preference was calculated as follows: [Sucrose intake (g)/(Sucrose + Water intake (g))] × 100%.

*Social Interaction Test*

According to previous studies (Cai et al., 2024; Jiang et al., 2019; Liu et al., 2020), social avoidance was assessed in a two-stage test conducted in an open-field arena (42 × 42 cm). In the first 5-minute “target absent” phase, the test mouse explored the arena containing an empty wire mesh cage. In the subsequent 5-minute “target present” phase, an unfamiliar CD1 mouse was placed in the cage. The time spent in the interaction zone (a 14 cm × 26 cm area surrounding the cage) during each phase was recorded using an automated tracking system (EthoVision XT, Noldus).

**References**

Cai, X.M., Sun, X.Y., Li, R., Wang, P.J., Qiu, J.C., Ge, Y.X., Yang, L., 2024. The hippocampal salt-inducible kinase 2-CREB-regulated transcription co-activator 1 system mediates the antidepressant actions of paroxetine in mice. Behav Brain Res 465, 114972.

Huang, J., Fan, H., Chen, Y.M., Wang, C.N., Guan, W., Li, W.Y., Shi, T.S., Chen, W.J., Zhu, B.L., Liu, J.F., Jiang, B., 2023. The salt-inducible kinases inhibitor HG-9-91-01 exhibits antidepressant-like actions in mice exposed to chronic unpredictable mild stress. Neuropharmacology 227, 109437.

Jiang, B., Wang, H., Wang, J.L., Wang, Y.J., Zhu, Q., Wang, C.N., Song, L., Gao, T.T., Wang, Y., Meng, G.L., Wu, F., Ling, Y., Zhang, W., Li, J.X., 2019. Hippocampal Salt-Inducible Kinase 2 Plays a Role in Depression via the CREB-Regulated Transcription Coactivator 1-cAMP Response Element Binding-Brain-Derived Neurotrophic Factor Pathway. Biol Psychiatry 85, 650-666.

Liu, Y., Tang, W., Ji, C., Gu, J., Chen, Y., Huang, J., Zhao, X., Sun, Y., Wang, C., Guan, W., Liu, J., Jiang, B., 2020. The Selective SIK2 Inhibitor ARN-3236 Produces Strong Antidepressant-Like Efficacy in Mice via the Hippocampal CRTC1-CREB-BDNF Pathway. Front Pharmacol 11, 624429.

Tian, X., Wang, G., Teng, F., Xue, X., Pan, J., Mao, Q., Guo, D., Song, X., Ma, K., 2024. Zhi Zi Chi decoction (Gardeniae fructus and semen Sojae Praeparatum) attenuates anxious depression via modulating microbiota-gut-brain axis in corticosterone combined with chronic restraint stress-induced mice. CNS Neurosci Ther 30, e14519.

Zhao, F., Piao, J., Song, J., Geng, Z., Chen, H., Cheng, Z., Cui, R., Li, B., 2025. Traditional Chinese herbal formula, Fuzi-Lizhong pill, produces antidepressant-like effects in chronic restraint stress mice through systemic pharmacology. J Ethnopharmacol 338, 119011.
